# Child–Pugh Score and *ABCG2*-rs2231142 Genotype Independently Predict Survival in Advanced Hepatoma Patients Treated with Sorafenib

**DOI:** 10.3390/jcm11092550

**Published:** 2022-05-02

**Authors:** Po-Han Huang, Jen Yu, Yin-Yi Chu, Yang-Hsiang Lin, Chau-Ting Yeh

**Affiliations:** 1 Department of Gastroenterology and Hepatology, New Taipei Municipal TuCheng Hospital, New Taipei 236, Taiwan; cksai@cgmh.org.tw (P.-H.H.); cyy2235@cgmh.org.tw (Y.-Y.C.); 2 College of Medicine, Chang Gung University, Taoyuan 333, Taiwan; 3 Department of Internal Medicine, Linkou Chang Gung Memorial Hospital, Taoyuan 333, Taiwan; joey20041010@gmail.com; 4 Liver Research Center, Linkou Chang Gung Memorial Hospital, Taoyuan 333, Taiwan; 5 Department of Gastroenterology and Hepatology, Linkou Chang Gung Memorial Hospital, Taoyuan 333, Taiwan

**Keywords:** hepatocellular carcinoma, prognostic factor, albumin-bilirubin grade, Child–Pugh score, genotypes

## Abstract

Patients with advanced hepatocellular carcinoma (HCC) are treated by immunotherapy and/or targeted agents, such as sorafenib. Several single nucleotide polymorphisms (SNPs) and clinical scores have been proposed as prognostic markers in HCC patients treated with sorafenib. This study aimed to validate the prognostic values of these markers in a tertiary referral medical center. Two independent cohorts (cohort-1 [*n* = 97] and cohort-2 [*n* = 60]) of advanced HCC patients treated with sorafenib monotherapy were enrolled. Univariate followed by multivariate Cox proportional hazard analysis identified Child–Pugh (CP) score (*p* < 0.001) and renal insufficiency during treatment (*p* < 0.001) as independent predictors in cohort-1 patients. The same analytic method revealed ascites (*p* = 0.000), CP score (*p* = 0.001), infection during treatment (*p* < 0.001), and ATP-binding cassette subfamily G member 2 (ABCG2)-rs2231142 genotype (*p* = 0.003) as independent predictors in cohort-2 patients. *ABCG2*-rs2231142 genotype “CC” was associated with unfavorable overall survival in sorafenib-treated HCC patients. In conclusion, the CP score and *ABCG2*-rs2231142 genotype served as independent survival predictors for advanced HCC patients receiving sorafenib treatment.

## 1. Introduction

Hepatocellular carcinoma (HCC) is one of the leading causes of cancer-related death worldwide [1]. The development of HCC is closely related to the underlying chronic liver diseases [2]. Alcohol abuse and chronic viral infections (hepatitis B and hepatitis C) are among the major causative etiologies, although their proportional distribution varies across regions and countries [2]. In Taiwan, during the period of 2003–2011, the age-standardized incidence rate of HCC was 32.97 per 100,000 person-years [3]. A marked decrease in the incidence rate was found in children (−16.6%) and young adults (−7.9%) during this period, whereas only a mild reduction was found in the middle-aged group (−2%). This decrease is largely attributed to the launch of a universal hepatitis B vaccination program.

The prognosis of patients with HCC is related to multiple factors. According to the most widely used practice guidance by the American Association for the Study of Liver Diseases, the number, size, and location of tumors, vascular invasion, distant metastasis, hepatic functional reserve, and patient performance status are crucial in HCC staging and prognosis [4]. Child–Pugh (CP) classification is the most widely used prognostic tool to assess liver function in HCC patients at present. This classification was originally developed to select cirrhotic patients with portal hypertension for elective variceal surgery [5,6]. In recent years, Johnson et al. developed a new HCC prognostic model, the albumin-bilirubin (ALBI) grade, which is simpler in terms of calculation and is based on two objective parameters: serum bilirubin levels and serum albumin levels [7]. It has been proposed that ALBI grade analysis, as well as CP classification, for the determination of clinical outcomes in HCC patients can be integrated into the Barcelona Clinic Liver Cancer (BCLC) system [8].

Sorafenib, a multityrosine kinase inhibitor, has become the first evidence-based systemic treatment regimen for HCC other than systemic cytotoxic chemotherapy since 2007 [9]. It remained the only choice for over a decade until multiple treatment options emerged in recent years. Of the newer systemic therapy regimens, the combination of atezolizumab and bevacizumab is the most promising treatment [10]. In Taiwan, sorafenib use for unresectable HCC has been financially supported by the National Health Insurance (NHI) since 2012 if HCC patients with Child–Pugh A have one of the following criteria: (1) extrahepatic metastasis, (2) major vascular invasion, or (3) insufficient response after ≥3 consecutive transcatheter arterial chemoembolizations (TACE). However, HCC patients are allowed to use sorafenib if they cover the cost themselves.

Increasing evidence supports that single nucleotide polymorphisms (SNPs) serve as prognostic markers in cancer progression. Previous studies reported that vascular endothelial growth factor receptor 2 (VEGFR2) rs7692791, WW domain-containing oxidoreductase (WWOX) rs9926344, carbonic anhydrase IX (CA9) rs1048638, and DICER rs1057035 polymorphisms were positively associated with poor prognosis in HCC patients [11,12,13,14]. Moreover, the SNPs of ATP-binding cassette subfamily B member 1 (ABCB1) and ATP-binding cassette subfamily G member 2 (ABCG2) were also clinically relevant in HCC progression [15,16,17]. Accordingly, in this study, we aimed to analyze the associations between these SNPs and clinical variables, including the CP score and ALBI score/grade, and survival in HCC patients treated with sorafenib.

## 2. Materials and Methods

### 2.1. Patients

From January 2015 to March 2018, 97 consecutive patients (cohort-1) with advanced HCC treated by sorafenib monotherapy in Chang Gung Memorial Hospital, Linkou branch, were enrolled. Patients received sorafenib at a target dose of 400 mg twice daily, and dose reduction and interruption were allowed. When tumor progression was achieved, sorafenib withdrawal and switching to other treatment modalities were allowed. All clinical parameters were collected retrospectively from medical records, including age, sex, alcohol history, tumor status, laboratory test prior to sorafenib treatment, previous and subsequent anti-HCC treatment, and adverse events during sorafenib use (Table 1). In an independent cohort (cohort-2), a total of 60 advanced HCC patients treated with sorafenib monotherapy were enrolled from 2007 to 2017. HCC patients were treated in the Chang Gung Memorial Hospital, Linkou and Keelung branches (from a different study group). The clinical characteristics of the 60 patients (cohort-2) are shown in Table 1. Cohort-2 samples had blood samples retrievable from Serum Bank, Chang Gung Memorial Hospital. Written informed consent was obtained from patients providing samples. The retrospective study was approved by the Institutional Review Board of Chang Gung Memorial Hospital, Linkou, Taiwan (IRB: 100–4426A3 and 104–168A3).

The diagnosis of HCC was made by dynamic computed tomography or dynamic magnetic resonance imaging. For tumors without a typical enhancing pattern by images, tissue proof was acquired. The ALBI score was calculated as (0.66 × log_10_ bilirubin [μmol/L]) + (–0.085) × albumin [g/L]), and the cutoff values of ALBI grades 1, 2, and 3 were ≤−2.60, between −2.60 and −1.39, and >−1.39, respectively [7]. Sorafenib-related adverse events were evaluated and graded according to Common Terminology Criteria for Adverse Events version 3.0, and all adverse events were managed promptly.

### 2.2. Genotyping

Genotyping of selected SNPs (VEGFR2 rs7692791, WWOX rs9926344, CA9 rs1048638, DICER rs1057035, ABCB1 rs2032582, ABCG2 rs2231142, and ABCG2 rs2231137) was performed using PCR and Sanger sequencing. Briefly, DNA from patient peripheral blood cells was extracted and purified using a FlexiGene DNA Kit (Qiagen, Valencia, CA, USA) according to the manufacturer’s instructions. The primers used in this study are listed below: VEGFR2 rs7692791-F: 5′-GCTATTGCAGAATAGGACAA-3′; VEGFR2 rs7692791-R: 5′-GTTGGAGAAAAGCTTGTCTT-3′, WWOX rs9926344-F: 5′-GAGTGTTCTGGGAGGCTGTT-3′; WWOX rs9926344-R: 5′-GCTGTACTTAAATAACCATGGCG-3′, CA9 rs1048638-F: 5′-TGTCCTGCTCATTATGCCACTTC-3′; CA9 rs1048638-R: 5′-GGGAACAAAGGTGACTAACACATATTT-3′, DICER rs1057035-F: 5′-TCTGCAGTTGCTTTTTCAAGACA-3′; DICER rs1057035-R: 5′-GAGACCGAATCTAATATGGAAAACCT-3′, ABCB1 rs2032582-F: 5′-GAAAATGTTGTCTGGACAAGC-3′; ABCB1 rs2032582-R: 5′-CATATTTAGTTTGACTCACC-3′, ABCG2 rs2231142-F: 5′-GGAGTTAACTGTCATTTGCA-3′; ABCG2 rs2231142-R: 5′-CTTGGAGTCTGCCACTTTAT-3′, and ABCG2 rs2231137-F: 5′-TTATCACAATGGTATGGGCC-3′; ABCG2 rs2231137-R: 5′-CTTTCTCAACTGGTTTTCGA-3′. CLC Sequence Viewer software was applied for SNP detection and analysis.

### 2.3. Statistical Analysis

The results are presented as case numbers and percentages for categorical variables and means ± standard deviations for continuous variables with normal distributions. For results not normally distributed, data are presented as the median (range). To examine factors associated with overall survival, we used univariate Cox proportional hazard models to calculate hazard ratios (HRs). All parameters showing statistical significance in the univariate analysis were included for multivariate analysis using the stepwise forward mode. This mode excludes nonsignificant parameters during the stepwise model building. As such, only significant parameters remained. A *p*-value < 0.05 was considered statistically significant. All statistical analyses were conducted by using Statistical Package for the Social Sciences (SPSS version 20, IBM Corp., Armonk, NY, USA).

## 3. Results

### 3.1. Baseline Characteristics

The clinical characteristics of the 97 patients in cohort-1 and 60 patients in cohort-2 are shown in Table 1. Notably, in cohort-1, patients with liver cirrhosis (85.6%), Child–Pugh B/C patients (20.7%), portal vein thrombosis (61.2%), metastasis before sorafenib treatment (55.7%), and ascites (24.7%) are shown in Table 1. On the other hand, in cohort-2, patients with liver cirrhosis (86.7%), Child–Pugh B/C patients (40%), portal vein thrombosis (66.7%), metastasis before sorafenib treatment (53.3%), and ascites (25%) are also listed in Table 1. The most common treatment-related adverse events were skin rash (13.4%) in cohort-1 and diarrhea (31.7%) in cohort-2. Data from cohort-1 and -2 patients were independently collected. Comparison of the clinical characteristics between cohort-1 and -2 patients showed that cohort-1 had a lower percentage of hepatitis C patients (*p* = 0.0189), a higher percentage of Child–Pugh stage A patients (*p* = 0.0233), a lower proportion of patients who developed distant metastasis during treatment (*p* = 0.0027), and a lower proportion of patients who experienced diarrhea during treatment (*p* < 0.001).

### 3.2. CP Score Acts as an Independent Factor for HCC Patients Treated with Sorafenib

**Figure 1:**
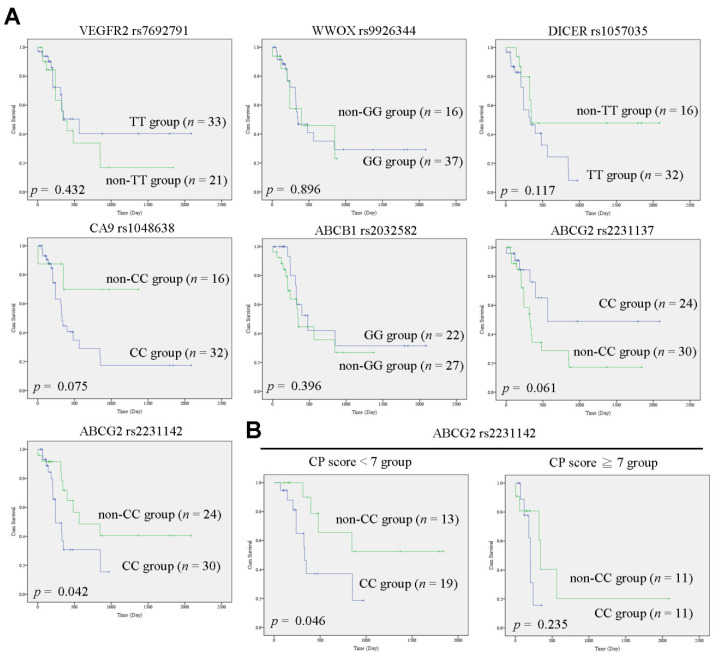
The ABCG rs2231142 polymorphism is correlated with the overall survival of HCC patients receiving sorafenib. (**A**) The clinical significances of VEGFR2 rs7692791, WWOX rs9926344, CA9 rs1048638, DICER rs1057035, ABCB1 rs2032582, ABCG2 rs2231142, and ABCG2 rs2231137 were calculated using Kaplan–Meier curves with log-rank analysis. (**B**) The clinical significance of ABCG2 rs2231142 in the CP score < 7 or ≥7 group is shown.

Cox proportional hazard analysis results for overall survival for cohort-1 patients are shown in Table 2. Univariate analysis revealed that HBsAg positivity, ascites, CP score, Eastern Cooperative Oncology Group (ECOG) performance status, serum bilirubin levels, and adverse events, including fatigue, renal insufficiency, and infection, were associated with overall survival. In the multivariate analysis, CP score (HR = 2.003, 95% CI = 1.455–2.758, *p* < 0.001) and renal insufficiency (HR = 7.661, 95% CI = 3.283–17.880, *p* < 0.001) remained independent predictors for survival in HCC patients. To further confirm that CP score served as an independent predictor in HCC patients with sorafenib treatment, the clinical significances between clinical parameters and survival outcomes in cohort-2 patients were analyzed (Table 3). As mentioned above, SNP genotypes have been used as prognostic markers in cancer progression. A literature search identified seven SNPs that were associated with HCC prognosis. Therefore, SNP genotyping for VEGFR2 rs7692791, WWOX rs9926344, CA9 rs1048638, DICER rs1057035, ABCB1 rs2032582, ABCG2 rs2231142, and ABCG2 rs2231137 was performed by PCR and Sanger sequencing. The prevalence of those SNPs in cohort-2 (*n* = 60) patients is shown in Table 4. In Kaplan–Meier analysis, we found that only ABCG2 rs2231142 genotype “CC” was significantly associated with poorer overall survival (Figure 1A). Specifically, the ABCG2 rs2231142 genotype “CC” group was significantly associated with poorer overall survival in CP score < 7 but not in CP score ≥ 7 (Figure 1B). Univariate Cox proportional hazard analysis in cohort-2 patients revealed that ascites (HR = 4.945, 95% CI = 2.019–12.108, *p* < 0.001), CP score (HR = 2.619, 95% CI = 1.154–5.945, *p* = 0.021), bilirubin (HR = 1.380, 95% CI = 1.105–1.723, *p* = 0.004), adverse events including hepatotoxicity (HR = 3.662, 95% CI = 1.056–12.695, *p* = 0.041), fatigue (HR = 3.738, 95% CI = 1.086–12.865, *p* = 0.037), renal insufficiency (HR = 3.853, 95% CI = 1.404–10.578, *p* = 0.009), infection (HR = 3.530, 95% CI = 1.361–9.157, *p* = 0.009), and ABCG2 rs2231142 genotypes (HR = 0.423, 95% CI = 0.181–0.992, *p* = 0.048) were associated with overall survival (Table 3). In multivariate analysis, ascites (HR = 9.947, 95% CI = 3.142–31.491, *p* = 0.000), CP score (HR = 5.776, 95% CI = 2.031–16.426, *p* = 0.001), infection (HR = 12.168, 95% CI = 3.680–40.231, *p* < 0.001), and ABCG2 rs2231142 (HR = 0.234, 95% CI = 0.090–0.607, *p* = 0.003) remained independent predictors for survival (Table 3). These findings supported that the CP score was a good predictor of overall survival for HCC patients treated with sorafenib.

## 4. Discussion

The ALBI grade has become an alternative HCC prognostic marker in recent years, especially for HCC patients treated with sorafenib. The superior prognostic value of ALBI grade compared with CP grade was reported among different treatment modalities of HCC, including liver resection [18], TACE [19], radiotherapy [20,21], and systemic treatment [22,23,24]. The ALBI grade has several advantages. First, the ALBI score is composed of two objective parameters. In contrast, the scoring of ascites and hepatic encephalopathy in CP classification is relatively subjective and lacks a universal and/or rigid definition. Second, the ALBI model is statistical-based, but the cutoff value of the CP score was arbitrarily defined in the beginning. As such, there is uncertainty for patients with CP scores immediately below or above the cutoff values. Third, in the CP score, the five parameters included are equally weighted, and some of them represent the same function. For instance, serum albumin and prothrombin time both represent hepatic synthetic function.

In this study, we found that the CP score served as a better clinical predictor of overall survival in HCC patients treated with sorafenib. In a large retrospective multicenter study, European HCC patients receiving sorafenib treatment were investigated [23]. Although the prognostic values of CP and ALBI were similar in the CP class A population, the CP score was likely to be more informative than ALBI in the overall population. In a Japanese study including 2584 treatment-naïve HCC patients, the ALBI grade was more discriminative for patients with better liver function [25]. Furthermore, the ALBI score was able to predict good versus poor prognosis in patients with a CP score of 5 [22,24]. Taken together, the ALBI score appeared to function better than the CP score in patients with good hepatic reserve, but it was not a satisfactory predictor for those with relatively poor liver function. In the present study, we had a higher proportion of patients who presented with ascites and a higher proportion of patients with a CP score > 6.

The scoring of ascites in CP classification was considered to be subjective and interrelated with serum albumin levels. However, the causes of ascites formation in patients with HCC are complex, including reduced osmotic pressure by hypoalbuminemia, portal hypertension caused by either cirrhotic liver or tumor vascular invasion, and peritoneal tumor seeding. In a retrospective study of patients with HCC treated with the combination therapy of TACE and sorafenib, the ALBI grade was more discriminative than the CP score, but its predictive value was not significant in the subgroup of portal vein tumor thrombosis [19]. Kim et al. compared the prognostic value of four liver function models in HCC patients with ascites. By integrating serum sodium levels into the model for end-stage liver disease (MELD) score, the MELD-Na score became a better outcome predictor for HCC patients with worsening liver function compared with the CP and ALBI scores [26]. A correlation was noted between cirrhotic ascites and serum sodium levels. Our study included patients with more advanced HCC, 61.2% with portal vein thrombosis, 55.7% with initial distant metastasis, and 24.7% with ascites. It is reasonable that the CP score became a better predictor of survival than the ALBI score in the study.

An association study between SNPs and drug responses in cancer progression has been reported. Scartozzi and coworkers demonstrated that VEGF rs2010963, VEGFR rs4604006, and BCLC stage served as predictors of overall survival and progression-free survival for HCC patients treated with sorafenib in Italy. Another study reported that VEGF rs1570360 and VEGFR2 rs2239702 were significantly associated with the sorafenib response in renal cell carcinoma [27]. Moreover, VEGF and ABCB1 polymorphisms correlated with sorafenib-mediated toxicity. The associations between the gene genotypes in the EGFR signaling pathway and HCC progression were determined [11]. The results showed that HCC patients with the VEGFR2 rs7692791 TT genotype were significantly correlated with poorer clinical outcomes. Another study demonstrated that HCC patients with the WWOX rs9926344 AA + AG genotypes had poorer survival outcomes by Kaplan–Meier curve analysis [12]. Furthermore, multivariate Cox regression analysis indicated that the WWOX rs9926344 AA + AG genotypes served as an independent predictor for recurrence-free survival in HCC patients. HCC patients with Dicer rs1057035 CT and CC genotypes had a better survival outcome than those with the Dicer rs1057035 TT genotype [14]. A report revealed that HCC patients carrying the CA9 rs1048638 CA genotype had poorer overall survival and disease-free survival than those carrying the CA9 rs1048638 CC genotype [13]. The distribution of ABCG2 rs2231142 genotypes in the Taiwanese population has been analyzed in the Taiwan Biobank database (https://taiwanview.twbiobank.org.tw/index accessed on 28 April 2022). The data showed that the frequency of the ABCG2 rs2231142 GG genotype (or CC genotype if named by the complementary strand) in a Taiwanese population (*n* = 1517) was approximately 68%. This prevalence was slightly greater than what was found in our study (Table 4, 53.3%). In the available SNP database (NCBI) or Tawain Biobank database, the reference SNP of ABCG2 rs2231142 is the G allele. However, in our and previous studies [15,17] investigating the correlation between ABCG2 rs2231142 genotypes and drug responses, the ABCG2 rs2231142 GG genotypes were referred to as the CC genotypes (named by the complementary strand). In this study, we followed the nomenclature of these relevant studies. ABCG2 functions as a transporter to regulate the efflux of drugs in cancer cells [28]. Kurdish breast cancer patients with ABCG2 rs2231142 AA exhibited a better response to anthracycline and paclitaxel treatment [29]. Consistently, in breast cancer, the ABCG2 rs2231142 AA group also had a better therapeutic response in patients receiving anthracycline chemotherapy [30]. Previous studies demonstrated that the ABCG2 rs2231142 polymorphism was associated with sorafenib and lenvatinib exposure [15,17]. A study investigated the associations between the ABCG2 rs2231142 polymorphism and lenvatinib pharmacokinetics (maximum plasma concentration [C_max_] and area under the plasma concentration curve [AUC]). In a study with a small sample size (*n* = 37), the genotypes of ABCG2 rs2231142 in Japanese HCC patients receiving lenvatinib exposure on Day 15 were analyzed. The results showed that the AUC and C_max_ on Day 15 in the ABCG2 rs2231142 non-CC group were significantly higher than those in the ABCG2 rs2231142 CC group [17]. Another report investigated the association between ABCG1 and ABCG2 genotypes and the sorafenib response in HCC patients [15]. The data indicated that the sorafenib concentration (dose/body weight) ratio was lower in the ABCG1 rs2032582 CT genotype compared to that in the ABCG1 rs2032582 CC or TT genotype. In addition, the sorafenib concentration (dose/body weight) ratio in HCC patients carrying the ABCG2 rs2231142 CA genotype was significantly lower than that in the ABCG2 rs2231142 CC group. HCC patients carrying the ABCG2 rs2231137 AG genotype had a significantly lower sorafenib concentration (dose/body weight) ratio than those carrying the ABCG2 rs2231137 GG genotype. These findings suggested that genetic polymorphisms, including those of ABCG1 and ABCG2, were correlated with distinct sorafenib treatment responses. In our study, only the ABCG2 rs2231142 genotype was correlated with survival outcome. In particular, sorafenib-treated HCC patients with the ABCG2 rs2231142 CC genotype had shorter overall survival. Based on our and previous studies, we hypothesized that the sorafenib response rate in HCC patients with the ABCG2 rs2231142 CC genotype was lower (than that in HCC patients with the non-CC genotype) due to a functionally more effective ABCG2 efflux transporter, leading to shorter overall survival. Recently, a group reported that ABCG2 rs2231142 genotypes combined with alcohol consumption might lead to increased HUA risk [31]. Taken together, genotypic polymorphisms of the efflux transporter genes, such as ABCG2 rs2231142 genotypes, play an important role in regulating HCC progression in Taiwanese HCC patients treated with targeted drugs.

The limitations of our study are its retrospective nature and single-center nature. The sample size was small. However, we used two independent cohorts of patients to compensate for this insufficiency.

## 5. Conclusions

In conclusion, the CP score is a good clinical predictor for survival in advanced HCC patients treated with sorafenib. Additionally, the ABCG2 rs2231142 polymorphism could serve as a prognostic predictor independent of CP score.

## Figures and Tables

**Table 1:** Basic clinical data of cohort-1 (*n* = 97) and cohort-2 (*n* = 60) patients.

Clinical Parameters	Cohort 1 (*n* = 97)	Cohort 2 (*n* = 60)	*p* Value
Gender, male, *n* (%)	82 (84.5%)	51 (85.0%)	0.9374
Age, years, mean ± SD	58.6 ± 10.6	58.1 ± 9.7	0.7834
ECOG status			0.9936
Status 0, *n* (%)	53 (54.6%)	34 (56.7%)	
Status 1, *n* (%)	37 (38.1%)	23 (38.3%)	
Status 2, *n* (%)	4 (4.1%)	2 (3.3%)	
Status 3, *n* (%)	3 (3.1%)	1 (1.7%)	
HBsAg, positive, *n* (%)	57 (58.8%)	38 (63.3%)	0.6166
Anti-HCV, positive, *n* (%)	30 (30.9%)	30 (50.0%)	**0.0189**
Alcoholism, yes, *n* (%)	33 (34%)	23 (38.3%)	0.6101
Cirrhosis, yes, *n* (%)	83 (85.6%)	52 (86.7%)	1.0000
Ascites, yes, *n* (%)	24 (24.7%)	15 (25.0%)	1.0000
Child–Pugh Score			**0.0233**
5–6, *n* (%)	77 (79.4%)	36 (60.0%)	
7–9, *n* (%)	18 (18.6%)	23 (38.3%)	
≥10, *n* (%)	2 (2.1%)	1 (1.7%)	
ALBI score, median (range)	−2.341 (−3.67 to −0.30)	−2.487 (−3.614 to −0.8922)	0.1225
ALBI grade			0.2156
Grade 1, *n* (%)	28 (28.9%)	20 (33.3%)	
Grade 2, *n* (%)	61 (62.9%)	39 (65.0%)	
Grade 3, *n* (%)	8 (8.2%)	1 (1.7%)	
Portal vein thrombosis, yes, *n* (%)	61 (61.2%)	40 (66.7%)	0.7321
Initial metastasis, yes, *n* (%) ^a^	54 (55.7%)	32 (53.3%)	0.8691
New metastasis, yes, *n* (%) ^b^	8 (8.2%)	16 (26.7%)	**0.0027**
All metastasis, yes, *n* (%) ^c^	62 (63.9%)	48 (80%)	**0.0225**
Tumor size, cm, median (range)	5.0 (1 to 18.6)	4.45 (1 to 17.5)	0.5872
Laboratory test			
AFP, ng/mL, median (range)	744.7 (2 to 831318)	1033 (4 to 745879)	0.4700
Albumin, g/L, mean ± SD	3.8 ± 0.5	3.807 ± 0.5276	0.5106
Bilirubin, mg/dL, mean ± SD	1.4 ± 2.2	1.330 ± 1.427	0.6429
Prothrombin time, sec, mean ± SD	13.1 ± 1.2	13.07 ± 1.083	0.8637
Creatinine, mg/dL, mean ± SD	0.8 ± 0.4	0.8492 ± 0.3740	0.5820
AST, U/L, mean ± SD	80.8 ± 61.0	82.4 ± 56.86	0.5941
ALT, U/L, mean ± SD	52.8 ± 37.3	57 ± 42.33	0.5865
Hemoglobin, g/dL, mean ± SD	12.5 ± 1.9	12.7 ± 1.745	0.4141
Platelet, 1000/μL, mean ± SD	161.7 ± 100.0	149.6 ± 90.06	0.4556
WBC, 1000/μL, mean ± SD	5.9 ± 2.6	5.663 ± 2.719	0.4788
Previous treatment, *n* (%)	76 (78.4%)	46 (76.7%)	0.8449
Sorafenib-related adverse events ^d^			
Leukopenia, *n* (%)	2 (2.1%)	1 (1.7%)	1.000
Neutropenia, *n* (%)	3 (3.1%)	1 (1.7%)	1.000
Anemia, *n* (%)	3 (3.1%)	2 (3.3%)	1.000
Thrombocytopenia, *n* (%)	12 (12.4%)	8 (13.3%)	1.000
Nausea, *n* (%)	0	0	
Vomiting, *n* (%)	0	0	
Mucositis, *n* (%)	1 (1%)	1 (1.7%)	1.000
Diarrhea, *n* (%)	4 (4.1%)	19 (31.7%)	**<0.001**
Alopecia, *n* (%)	0	0	
Hepatotoxicity, *n* (%)	5 (5.2%)	3 (5.0%)	1.000
Skin rash, *n* (%)	13 (13.4%)	7 (11.7%)	0.8104
Fatigue, *n* (%)	3 (3.1%)	3 (5.0%)	0.6752
Renal insufficiency, *n* (%) ^e^	8 (8.2%)	6 (10.0%)	0.7764
Bleeding, *n* (%)	6 (6.2%)	6 (10.0%)	0.5378
Infection, *n* (%)	11 (11.3%)	7 (11.7%)	1.000

^a^ Initial metastasis detected before sorafenib treatment; ^b^ distant extrahepatic metastasis detected during/after sorafenib treatment; ^c^ all metastasis before mortality; ^d^ ≥grade 3; ^e^ all renal failures ≥ grade 3 were caused by sepsis; AFP, alpha-fetoprotein; AST, aspartate transaminase; ALT, alanine transaminase; WBC, white blood cell; ECOG, Eastern Cooperative Oncology Group; HBsAg, hepatitis B virus surface antigen; HCV, hepatitis C virus.

**Table 2:** Cox proportional hazard analysis for association between clinical factors and overall survival (cohort-1).

Clinical Parameters	Univariate Analysis		Multivariate Analysis ^a^	
	Hazard Ratio (95% CI)	*p*	Hazard Ratio (95% CI)	*p*
Gender, male = 1	1.442 (0.607–3.428)	0.407		
Age, per year increase	1.011 (0.981–1.041)	0.478		
Anti-HCV, positive = 1	2.018 (0.988–4.123)	0.054		
HBsAg, positive = 1	1.914 (1.013–3.618)	**0.046**		
Alcoholism, yes = 1	1.103 (0.576–2.111)	0.768		
Cirrhosis, yes = 1	1.213 (0.373–3.947)	0.748		
Ascites, yes = 1	2.884 (1.458–5.703)	**0.002**		
Child–Pugh Score, per score increase	1.754 (1.285–2.393)	**<0.001**	2.003 (1.455–2.758)	**<0.001**
Child–Pugh Score, “≥7” = 1	3.895 (1.799–8.435)	**0.001**		
ALBI score, per score increase	1.536 (0.848–2.782)	0.157		
ECOG, per score increase	1.577 (1.069–2.327)	**0.022**		
Portal vein thrombosis, yes = 1	1.800 (0.959–3.381)	0.067		
All metastasis, yes = 1	1.339 (0.695–2.579)	0.383		
Initial metastasis, yes = 1	1.000 (0.547–1.827)	1.000		
New metastasis after regression, yes = 1	1.709 (0.818–3.567)	0.154		
Tumor size, per cm increase	1.002 (0.927–1.083)	0.963		
Alpha-fetoprotein, per 1000 ng/mL increase	1.001 (0.999–1.004)	0.300		
Albumin, per g/L increase	0.539 (0.282–1.029)	0.061		
Bilirubin, per mg/dL increase	1.240 (1.029–1.494)	**0.024**		
Prothrombin time, per s increase	0.749 (0.559–1.004)	0.053		
Creatinine, per mg/dL increase	1.247 (0.745–2.087)	0.401		
AST, per U/L increase	1.003 (0.995–1.010)	0.486		
ALT, per U/L increase	0.990 (0.979–1.002)	0.106		
Hb, per g/dL increase	0.919 (0.777–1.087)	0.325		
Platelet, per 1000/μL increase	1.003 (0.999–1.006)	0.107		
WBC, per 1000/μL increase	1.029 (0.911–1.161)	0.648		
Neutrophil, per 1000/μL increase	1.040 (0.911–1.189)	0.560		
Neutrophil ratio	1.013 (0.989–1.039)	0.284		
Lymphocyte, per 1000/μL	0.946 (0.605–1.478)	0.807		
N to L ratio	0.970 (0.841–1.119)	0.677		
P to L ratio	1.000 (0.997–1.003)	0.955		
Previous treatment, yes = 1	1.185 (0.575–2.441)	0.645		
Adverse events ≥ grade 3, yes = 1				
Leukopenia	1.260 (0.172–9.238)	0.820		
Neutropenia	0.915 (0.220–3.804)	0.903		
Anemia	0.629 (0.086–4.587)	0.648		
Thrombocytopenia	1.251 (0.579–2.705)	0.569		
Nausea	-	-		
Vomiting	-	-		
Mucositis	0.049 (0–29808681482)	0.827		
Diarrhea	0.325 (0.076–1.386)	0.129		
Alopecia	-	-		
Hepatotoxicity	2.824 (0.989–8.064)	0.052		
Skin rash	0.720 (0.283–1.837)	0.492		
Fatigue	4.287 (1.305–14.081)	**0.016**		
Renal insufficiency	5.065 (2.283–11.233)	**<0.001**	7.661 (3.283–17.880)	**<0.001**
Bleeding	2.677 (0.949–7.555)	0.063		
Infection	3.860 (1.840–8.096)	**<0.001**		

^a^ Multivariate analysis was performed using stepwise forward mode. Bold letters, *p* < 0.05.

**Table 3:** Cox proportional hazard analysis for association between clinical factors and overall survival (cohort-2).

Clinical Parameters	Univariate Analysis		Multivariate Analysis ^a^	
	Hazard Ratio (95% CI)	*p*	Hazard Ratio (95% CI)	*p*
Gender, male = 1	1.442 (0.607–3.428)	0.407		
Age, >65 = 1	0.954 (0.323–2.814)	0.932		
Anti-HCV, positive = 1	0.434 (0.147–1.283)	0.131		
HBsAg, positive = 1	2.524 (0.930–6.851)	0.069		
Alcoholism, yes = 1	0.833 (0.343–2.021)	0.686		
Cirrhosis, yes = 1	1.520 (0.201–11.486)	0.685		
Ascites, yes = 1	4.945 (2.019–12.108)	**0.000**	9.947 (3.142–31.491)	**<0.001**
Child–Pugh Score, “≥ 7” = 1	2.619 (1.154–5.945)	**0.021**	5.776 (2.031–16.426)	**0.001**
ALBI score	2.460 (0.949–6.379)	0.064		
ECOG	1.335 (0.579–3.078)	0.497		
Portal vein thrombosis, yes = 1	1.204 (0.524–2.769)	0.662		
All metastasis, yes = 1	1.632 (0.982–2.711)	0.059		
Initial metastasis, yes = 1	1.096 (0.484–2.483)	0.826		
Tumor size, per cm increase	0.978 (0.875–1.092)	0.690		
Alpha-fetoprotein, per 1000 ng/mL increase	0.871 (0.384–1.977)	0.741		
Albumin, per g/L increase	0.782 (0.489–1.252)	0.306		
Bilirubin, per mg/dL increase	1.380 (1.105–1.723)	**0.004**		
Prothrombin time, per s increase	0.732 (0.480–1.116)	0.147		
Creatinine, per mg/dL increase	1.593 (0.602–4.215)	0.348		
AST, per U/L increase	1.002 (0.993–1.012)	0.642		
ALT, per U/L increase	0.992 (0.978–1.006)	0.264		
Hb, per g/dL increase	0.796 (0.616–1.028)	0.080		
Platelet, per 1000/μL increase	1.002 (0.997–1.006)	0.497		
WBC, per 1000/μL increase	1.020 (0.878–1.185)	0.796		
Previous treatment, yes = 1	1.328 (0.547–3.227)	0.531		
Adverse events ≥ grade 3, yes = 1				
Leukopenia	0.047 (0.000–2187.519)	0.577		
Neutropenia	0.044 (0.000–144.587)	0.450		
Anemia	0.048 (0.000–188,112)	0.190		
Vomiting	-	-		
Mucositis	0.048 (0.829–44,109,144,047)	0.829		
Alopecia	-	-		
Hepatotoxicity	3.662 (1.056–12.695)	**0.041**		
Skin rash	0.860 (0.201–3.683)	0.839		
Fatigue	3.738 (1.086–12.865)	**0.037**		
Renal insufficiency	3.853 (1.404–10.578)	**0.009**		
Bleeding	2.679 (0.902–7.952)	0.076		
Infection	3.530 (1.361–9.157)	0.009	12.168 (3.680–40.231)	**<0.001**
VEGFR2 rs7692791 non-TT = 1	1.377 (0.617–3.073)	0.435		
WWOX rs9926344 non-GG = 1	1.061 (0.439–2.565)	0.896		
DICER rs1057035 non-TT = 1	0.499 (0.205–1.212)	0.125		
CA9 rs1048638 non-CC = 1	0.288 (0.067–1.238)	0.094		
ABCB1 rs2032582 non-GG = 1	1.443 (0.615–3.384)	0.399		
ABCG2 rs2231142 non-CC = 1	0.423 (0.181–0.992)	**0.048**	0.234 (0.090–0.607)	**0.003**
ABCG2 rs2231137 non-CC = 1	2.361 (0.934–5.968)	0.069		

^a^ Multivariate analysis was performed using stepwise forward mode. Bold letters, *p* < 0.05.

**Table 4:** Frequency distributions of VEGFR2, WWOX, CA9, DICER, ABCB1, ABCG2, and ABCG2 polymorphisms in patients with HCC.

Gene	SNP	Types	
VEGFR2	rs7692791	TT type (*n* = 35, 58.3%)	non-TT type (*n* = 25, 41.7%)
WWOX	rs9926344	GG type (*n* = 43, 72.9%)	non-GG type (*n* = 16, 27.1%)
CA9	rs1048638	CC type (*n* = 52, 86.7%)	non-CC type (*n* = 8, 13.3%)
DICER	rs1057035	TT type (*n* = 34, 63%)	non-TT type (*n* = 20, 37%)
ABCB1	rs2032582	GG type (*n* = 24, 44.4%)	non-GG type (*n* = 30, 55.6%)
ABCG2	rs2231142	CC type (*n* = 32, 53.3%)	non-CC type (*n* = 28, 46.7%)
ABCG2	rs2231137	CC type (*n* = 26, 43.3%)	non-CC type (*n* = 34, 56.7%)

## Data Availability

Not applicable.
